# Crystal structure of cyclo­hexyl­ammonium thio­cyanate

**DOI:** 10.1107/S2056989014027297

**Published:** 2015-01-01

**Authors:** Abdulaziz A. Bagabas, Sultan B. Alhoshan, Hazem A. Ghabbour, C. S. Chidan Kumar, Hoong-Kun Fun

**Affiliations:** a National Petrochemical Technology Center (NPTC), Materials Science Research Institute (MSRI), King Abdulaziz City for Science and Technology (KACST), PO Box 6086, Riyadh 11442, Saudi Arabia; b Department of Pharmaceutical Chemistry, College of Pharmacy, King Saud University, PO Box 2457, Riaydh 11451, Saudi Arabia; c X-ray Crystallography Unit, School of Physics, Universiti Sains Malaysia, 11800 USM, Penang, Malaysia; d Department of Chemistry, Alva’s Institute of Engineering & Technology, Mijar, Moodbidri 574225, Karnataka, India

**Keywords:** crystal structure, cyclo­hexyl­ammonium, distorted chair, hydrogen bonding

## Abstract

In the title salt, C_6_H_11_NH_3_^+^·SCN^−^, the cyclo­hexyl­ammonium ring adopts a slightly distorted chair conformation. The ammonium group occupies an equatorial position to minimize 1,3 and 1,5 diaxial inter­actions. In the crystal, the components are linked by N—H⋯N and N—H⋯S hydrogen-bonding inter­actions, resulting in a three-dimensional network.

## Related literature   

For the synthesis and uses of the title compound, see: Baluja *et al.* (1960 ▸); Coddens *et al.* (1986 ▸); Goel (1988 ▸); Mathes *et al.* (1948 ▸) 1955 ▸); Mathes & Stewart (1955 ▸); Morrison & Ratcliffe (1953 ▸); Stewart (1951 ▸); For the structures of other cyclo­hexyl­ammonium salts, see: Bagabas *et al.* (2014 ▸); Shimada *et al.* (1955 ▸); Smith *et al.* (1994 ▸); Odendal *et al.* (2010 ▸).
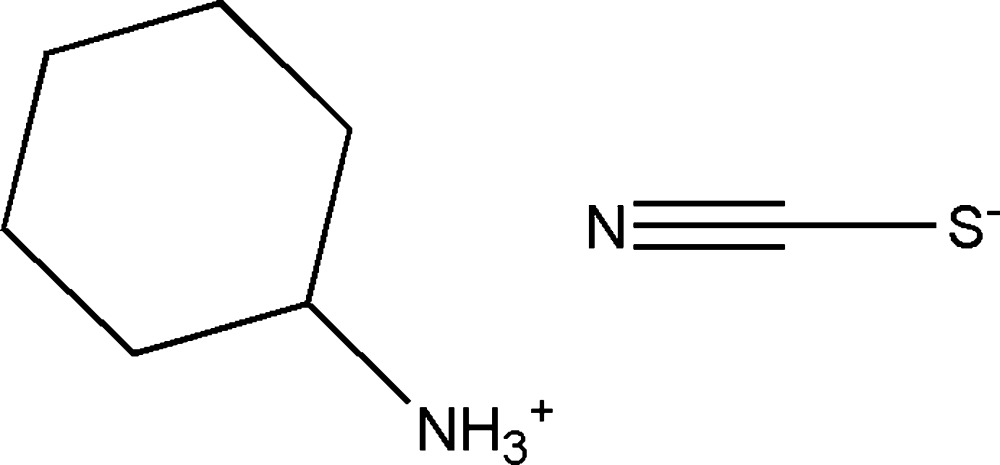


## Experimental   

### Crystal data  

C_6_H_14_N^+^·NCS^−^
*M* *_r_* = 158.26
Trigonal, 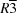

*a* = 23.4036 (6) Å
*c* = 8.3373 (2) Å
*V* = 3954.8 (2) Å^3^
*Z* = 18
Cu *K*α radiation
μ = 2.71 mm^−1^
*T* = 296 K
0.98 × 0.25 × 0.11 mm

### Data collection  

Bruker APEXII CCD diffractometer
Absorption correction: multi-scan (*SADABS*; Bruker, 2009 ▸) *T*_min_ = 0.176, *T*_max_ = 0.748
10355 measured reflections
1670 independent reflections
1530 reflections with *I* > 2σ(*I*)
*R*_int_ = 0.051

### Refinement  

*R*[*F* ^2^ > 2σ(*F* ^2^)] = 0.038
*wR*(*F* ^2^) = 0.100
*S* = 1.06
1670 reflections
103 parameters
H atoms treated by a mixture of independent and constrained refinement
Δρ_max_ = 0.22 e Å^−3^
Δρ_min_ = −0.28 e Å^−3^

### 

Data collection: *APEX2* (Bruker, 2009 ▸); cell refinement: *SAINT* (Bruker, 2009 ▸); data reduction: *SAINT*; program(s) used to solve structure: *SHELXS97* (Sheldrick, 2008 ▸); program(s) used to refine structure: *SHELXL2013* (Sheldrick, 2008 ▸); molecular graphics: *SHELXTL* (Sheldrick, 2008 ▸); software used to prepare material for publication: *SHELXTL* and *PLATON* (Spek, 2009 ▸).

## Supplementary Material

Crystal structure: contains datablock(s) I, global. DOI: 10.1107/S2056989014027297/sj5432sup1.cif

Structure factors: contains datablock(s) I. DOI: 10.1107/S2056989014027297/sj5432Isup2.hkl

Supporting information file. DOI: 10.1107/S2056989014027297/sj5432Isup3.cml

**Fig. 1.**
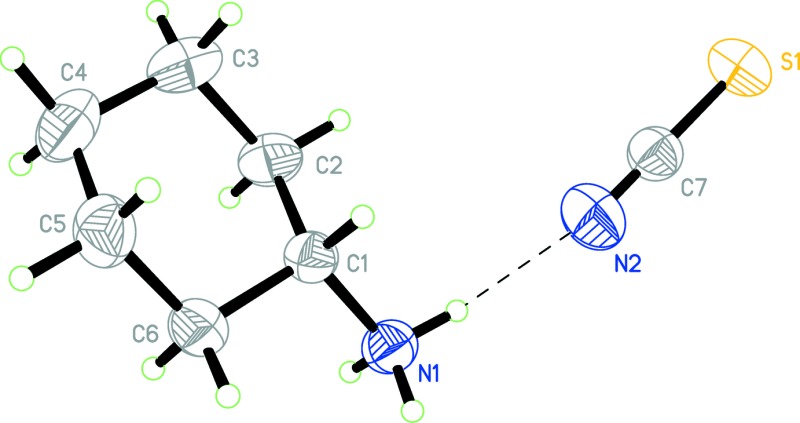
The molecular structure of the title compound with atom labels and 50% probability displacement ellipsoids. The strong N—H···N hydrogen bond linking the cation and the anion is shown as a dashed line.

. DOI: 10.1107/S2056989014027297/sj5432fig1.tif
The molecular structure of the title compound with atom labels and 50% probability displacement ellipsoids. The strong N—H⋯N hydrogen bond linking the cation and the anion is shown as a dashed line.

**Fig. 2.**
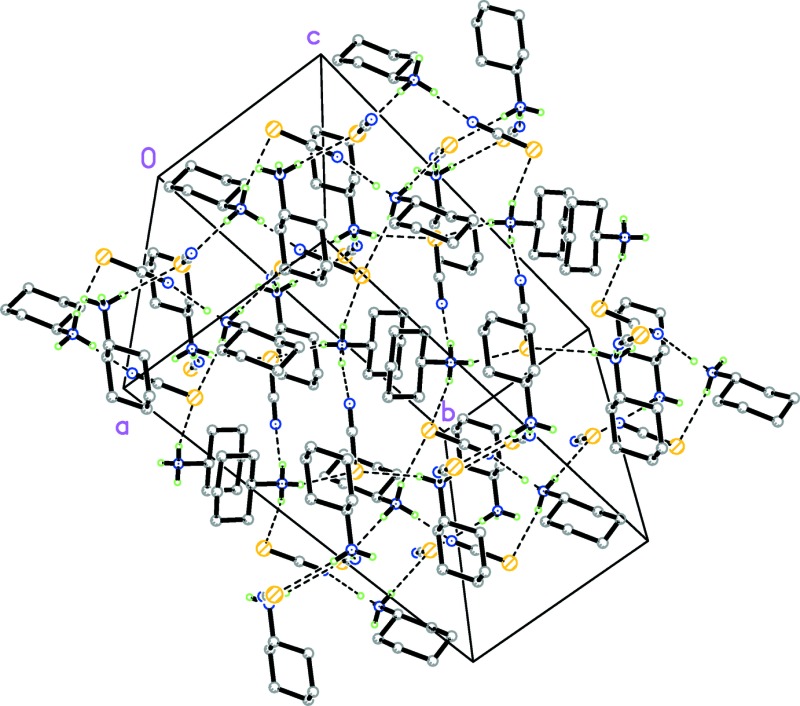
Crystal packing of the title compound, showing the N–H···N and N–H···S hydrogen bonding interactions (see Table 1) as dashed lines producing a three-dimensional network

. DOI: 10.1107/S2056989014027297/sj5432fig2.tif
Crystal packing of the title compound, showing the N–H⋯N and N–H⋯S hydrogen bonding inter­actions (see Table 1) as dashed lines producing a three-dimensional network

CCDC reference: 1039130

Additional supporting information:  crystallographic information; 3D view; checkCIF report

## Figures and Tables

**Table 1:** Hydrogen-bond geometry (, )

*D*H*A*	*D*H	H*A*	*D* *A*	*D*H*A*
N1H1*N*S1^i^	0.89(2)	2.62(2)	3.4955(14)	167(2)
N1H2*N*N2	0.90(2)	1.94(2)	2.822(2)	170.4(18)
N1H3*N*S1^ii^	0.87(2)	2.573(18)	3.4214(14)	165.5(19)

Symmetry codes: (i) 
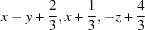
; (ii) 
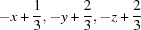
.

## supplementary crystallographic information

### S1. Chemical context

The title compound, C_6_H_11_NH_3_^+^.SCN^-^, has previously been synthesized by reacting an aqueous solution of cyclo­hexyl­amine (CHA) with ammonium thio­cyanate (H_4_N^+^SCN^-^) at 85–90 °C, followed by extraction of C_6_H_11_NH_3_^+^.SCN^-^ with benzene, and then recrystallization from ethanol (Mathes *et al.*, 1948). This compound is used as an animal repellent and also as an insecticide or fungicide (Stewart, 1951). It is also a starting material for the preparation of other compounds (Baluja *et al.*, 1960; Morrison *et al.*, 1953; Stewart, 1951), an accelerator and activator for rubber vulcanization (Mathes *et al.*, 1955) and an accelerator for the curing of polyepoxide-polyimine materials (Goel, 1988). It has also been used as a stationary phase for gas chromatography (Coddens *et al.*, 1986). Nevertheless, the crystal structure of this important compound has not been determined. We report here the crystal structure of C_6_H_11_NH_3_^+^.SCN^-^ together with a new room-temperature synthesis using a salt metathesis reaction. This is a simpler process and results in a higher yield than the one published in the literature. Furthermore, we aim to use this compound to prepare new metal complexes based on both the cyclo­hexyl­ammonium cation and the thio­cyanate anion.

### S2. Structural commentary

The assymmetric unit of the title compound (Fig. 1), contains one cyclo­hexyl­ammonium cation (C1–C6/N1) and one thio­cynate anion (S1/C7/N2). The cyclo­hexyl­ammonium ring adopts a slightly distorted chair conformation, with puckering parameters: Q = 0.5669 (18) Å, *θ* = 177.95 (18)°, and *φ* = 161 (5)°. For an ideal chair configuration, *θ* has a value of 0 or 180°. The ammonium functional group is at an equatorial position to minimize 1,3 and 1,5 di-axial inter­actions. The bond lengths and bond angles are in normal ranges and are comparable with those reported earlier for similar compounds (Bagabas *et al.*, 2014; Shimada *et al.*, 1955; Smith *et al.*, 1994; Odendal *et al.*, 2010).

### S3. Supra­molecular features

In the asymmetric unit, a strong N1–H2N···N2 hydrogen bond links the cation and the anion. In the crystal structure, these contacts are supported by inter­molecular N1–H1N···S1 and N1–H3N···S1 hydrogen bonds (Symmetry codes: *x*-*y*+2/3, *x*+1/3, -*z*+4/3; -*x*+1/3, -*y*+2/3, -*z*+2/3) with S1 as a bifurcated acceptor (Fig. 2) to produce a three-dimensional network.

### S4. Synthesis and crystallization

The title compound, C_6_H_11_NH_3_^+^.SCN^-^, was prepared by exchanging counter ions in a salt metathesis reaction between sodium thio­cyanate (NaSCN) and cyclo­hexyl­ammonium chloride (C_6_H_11_NH_3_^+. ^Cl^-^) in ethano­lic medium, where the precipitation of sodium chloride (NaCl) is the driving force for the reaction. In typical reaction, 100 mmol of NaSCN was dissolved in 350 ml absolute ethanol, while 100 mmol C_6_H_11_NH_3_^+^Cl^-^ was dissolved separately in 250 ml absolute ethanol. Combining these two solutions at room temperature resulted in white precipitate of NaCl, as confirmed by X-ray powder diffraction (PXRD), which was filtered off through F-size fritted filter. The filtrate was left for the solvent to evaporate to dryness at room temperature. About 200 ml absolute ethanol was added to the residual solid to dissolve the desired product, C_6_H_11_NH_3_^+^.SCN^-^. It was noticed at this step that a small amount of the residual solid did not dissolve and it was separated by filtration. This undissolved material was NaCl, again identified by PXRD. Slow evaporation at room temperature over a period of 10 days of the ethano­lic solution resluted in colorless crystals of C_6_H_11_NH_3_^+^.SCN^-^ (yield = around 100%) suitable for single crystal X-ray diffraction studies. It is advisable to recrystallize this light-sensitive material in the dark. The chemical composition of the desired product was also confirmed by C, H, N, S elemental microanalysis: (%C: 52.67 exp.; 53.11 cal.), (%H: 9.30 exp.; 8.93 cal.), (%N: 17.85 exp.; 17.70 cal.), and (%S: 20.18 exp.; 20.25 cal.). Potassium thio­cyanate (KSCN) can be used instead of NaSCN for executing the reaction, where KCl precipitates and C_6_H_11_NH_3_^+^.SCN^-^ produces with around 98% yield.

### S5. Refinement details

The nitro­gen-bound H-atoms were located in a difference Fourier map and were refined freely (NH = 0.87 (2), 0.90 (2) and 0.89 (2) Å). Other H atoms were positioned geometrically (C—H 0.97–0.98 Å) and refined using a riding model with *U*_iso_(H) = 1.2 *U*_eq_(C)

### Crystal data

**Table:**

C_6_H_14_N^+^·NCS^−^	*D*_x_ = 1.196 Mg m^−^^3^
*M**_r_* = 158.26	Cu *K*α radiation, λ = 1.54178 Å
Trigonal, *R*3:*H*	Cell parameters from 3689 reflections
*a* = 23.4036 (6) Å	θ = 3.8–71.3°
*c* = 8.3373 (2) Å	µ = 2.71 mm^−^^1^
*V* = 3954.8 (2) Å^3^	*T* = 296 K
*Z* = 18	Needle, colourless
*F*(000) = 1548	0.98 × 0.25 × 0.11 mm

### Data collection

**Table:**

Bruker APEXII CCD diffractometer	1530 reflections with *I* > 2σ(*I*)
φ and ω scans	*R*_int_ = 0.051
Absorption correction: multi-scan (*SADABS*; Bruker, 2009)	θ_max_ = 72.1°, θ_min_ = 3.8°
*T*_min_ = 0.176, *T*_max_ = 0.748	*h* = −28→28
10355 measured reflections	*k* = −28→28
1670 independent reflections	*l* = −7→9

### Refinement

**Table:**

Refinement on *F*^2^	0 restraints
Least-squares matrix: full	Hydrogen site location: mixed
*R*[*F*^2^ > 2σ(*F*^2^)] = 0.038	H atoms treated by a mixture of independent and constrained refinement
*wR*(*F*^2^) = 0.100	*w* = 1/[σ^2^(*F*_o_^2^) + (0.0622*P*)^2^ + 1.6831*P*] where *P* = (*F*_o_^2^ + 2*F*_c_^2^)/3
*S* = 1.06	(Δ/σ)_max_ < 0.001
1670 reflections	Δρ_max_ = 0.22 e Å^−^^3^
103 parameters	Δρ_min_ = −0.28 e Å^−^^3^

### Special details

**Table:**

Geometry. All e.s.d.'s (except the e.s.d. in the dihedral angle between two l.s. planes) are estimated using the full covariance matrix. The cell e.s.d.'s are taken into account individually in the estimation of e.s.d.'s in distances, angles and torsion angles; correlations between e.s.d.'s in cell parameters are only used when they are defined by crystal symmetry. An approximate (isotropic) treatment of cell e.s.d.'s is used for estimating e.s.d.'s involving l.s. planes.

### Fractional atomic coordinates and isotropic or equivalent isotropic displacement parameters (Å^2^)

**Table:**

	*x*	*y*	*z*	*U*_iso_*/*U*_eq_	
S1	0.06708 (2)	0.40416 (2)	0.39763 (4)	0.04932 (18)	
N1	0.22496 (6)	0.30544 (6)	0.62467 (16)	0.0396 (3)	
N2	0.15453 (8)	0.36894 (8)	0.5327 (2)	0.0628 (4)	
C1	0.17544 (6)	0.24495 (6)	0.71255 (15)	0.0339 (3)	
H1A	0.1541	0.2582	0.7939	0.041*	
C2	0.12316 (7)	0.19773 (7)	0.59728 (17)	0.0433 (3)	
H2A	0.1437	0.1862	0.5119	0.052*	
H2B	0.1005	0.2187	0.5496	0.052*	
C3	0.07361 (7)	0.13543 (8)	0.6863 (2)	0.0525 (4)	
H3A	0.0494	0.1465	0.7626	0.063*	
H3B	0.0422	0.1041	0.6102	0.063*	
C4	0.10721 (9)	0.10360 (7)	0.7747 (2)	0.0570 (4)	
H4A	0.1264	0.0871	0.6973	0.068*	
H4B	0.0746	0.0664	0.8364	0.068*	
C5	0.16092 (8)	0.15210 (8)	0.88648 (19)	0.0502 (4)	
H5A	0.1836	0.1312	0.9345	0.060*	
H5B	0.1410	0.1643	0.9721	0.060*	
C6	0.21055 (7)	0.21389 (7)	0.79664 (18)	0.0422 (3)	
H6A	0.2427	0.2452	0.8715	0.051*	
H6B	0.2339	0.2025	0.7184	0.051*	
C7	0.11856 (7)	0.38340 (7)	0.47760 (17)	0.0426 (3)	
H3N	0.2412 (9)	0.2942 (10)	0.545 (2)	0.055 (5)*	
H2N	0.2062 (9)	0.3280 (9)	0.587 (2)	0.046 (4)*	
H1N	0.2572 (10)	0.3321 (10)	0.691 (3)	0.061 (5)*	

### Atomic displacement parameters (Å^2^)

**Table:**

	*U*^11^	*U*^22^	*U*^33^	*U*^12^	*U*^13^	*U*^23^
S1	0.0485 (2)	0.0679 (3)	0.0414 (3)	0.03650 (19)	0.00383 (14)	0.00618 (15)
N1	0.0367 (6)	0.0371 (6)	0.0419 (7)	0.0162 (5)	−0.0010 (5)	0.0025 (5)
N2	0.0598 (8)	0.0673 (9)	0.0734 (10)	0.0408 (8)	−0.0064 (7)	0.0012 (7)
C1	0.0320 (6)	0.0350 (6)	0.0344 (7)	0.0165 (5)	0.0010 (5)	0.0007 (5)
C2	0.0362 (7)	0.0460 (7)	0.0398 (8)	0.0145 (6)	−0.0040 (5)	−0.0015 (6)
C3	0.0374 (7)	0.0488 (8)	0.0518 (9)	0.0070 (6)	0.0015 (6)	−0.0043 (7)
C4	0.0615 (9)	0.0362 (7)	0.0624 (10)	0.0162 (7)	0.0150 (8)	0.0050 (7)
C5	0.0567 (9)	0.0488 (8)	0.0500 (9)	0.0299 (7)	0.0048 (7)	0.0119 (6)
C6	0.0379 (7)	0.0437 (7)	0.0469 (8)	0.0218 (6)	−0.0034 (6)	0.0032 (6)
C7	0.0416 (7)	0.0437 (7)	0.0417 (8)	0.0207 (6)	0.0031 (6)	−0.0006 (6)

### Geometric parameters (Å, º)

**Table:**

S1—C7	1.6486 (15)	C3—C4	1.517 (3)
N1—C1	1.4978 (16)	C3—H3A	0.9700
N1—H3N	0.87 (2)	C3—H3B	0.9700
N1—H2N	0.90 (2)	C4—C5	1.520 (2)
N1—H1N	0.89 (2)	C4—H4A	0.9700
N2—C7	1.148 (2)	C4—H4B	0.9700
C1—C2	1.5132 (18)	C5—C6	1.524 (2)
C1—C6	1.5142 (18)	C5—H5A	0.9700
C1—H1A	0.9800	C5—H5B	0.9700
C2—C3	1.527 (2)	C6—H6A	0.9700
C2—H2A	0.9700	C6—H6B	0.9700
C2—H2B	0.9700		
			
C1—N1—H3N	109.8 (13)	C2—C3—H3B	109.2
C1—N1—H2N	110.7 (11)	H3A—C3—H3B	107.9
H3N—N1—H2N	108.9 (17)	C3—C4—C5	111.65 (13)
C1—N1—H1N	109.9 (13)	C3—C4—H4A	109.3
H3N—N1—H1N	110.1 (18)	C5—C4—H4A	109.3
H2N—N1—H1N	107.5 (16)	C3—C4—H4B	109.3
N1—C1—C2	109.91 (11)	C5—C4—H4B	109.3
N1—C1—C6	109.33 (11)	H4A—C4—H4B	108.0
C2—C1—C6	112.22 (11)	C4—C5—C6	111.14 (13)
N1—C1—H1A	108.4	C4—C5—H5A	109.4
C2—C1—H1A	108.4	C6—C5—H5A	109.4
C6—C1—H1A	108.4	C4—C5—H5B	109.4
C1—C2—C3	109.83 (11)	C6—C5—H5B	109.4
C1—C2—H2A	109.7	H5A—C5—H5B	108.0
C3—C2—H2A	109.7	C1—C6—C5	110.12 (11)
C1—C2—H2B	109.7	C1—C6—H6A	109.6
C3—C2—H2B	109.7	C5—C6—H6A	109.6
H2A—C2—H2B	108.2	C1—C6—H6B	109.6
C4—C3—C2	111.86 (13)	C5—C6—H6B	109.6
C4—C3—H3A	109.2	H6A—C6—H6B	108.2
C2—C3—H3A	109.2	N2—C7—S1	179.71 (16)
C4—C3—H3B	109.2		
			
N1—C1—C2—C3	178.72 (12)	C3—C4—C5—C6	−54.67 (18)
C6—C1—C2—C3	56.83 (16)	N1—C1—C6—C5	−179.85 (12)
C1—C2—C3—C4	−54.73 (17)	C2—C1—C6—C5	−57.63 (16)
C2—C3—C4—C5	54.43 (18)	C4—C5—C6—C1	55.66 (17)

### Hydrogen-bond geometry (Å, º)

**Table:**

*D*—H···*A*	*D*—H	H···*A*	*D*···*A*	*D*—H···*A*
N1—H1*N*···S1^i^	0.89 (2)	2.62 (2)	3.4955 (14)	167 (2)
N1—H2*N*···N2	0.90 (2)	1.94 (2)	2.822 (2)	170.4 (18)
N1—H3*N*···S1^ii^	0.87 (2)	2.573 (18)	3.4214 (14)	165.5 (19)

Symmetry codes: (i) *x*−*y*+2/3, *x*+1/3, −*z*+4/3; (ii) −*x*+1/3, −*y*+2/3, −*z*+2/3.
