# Supplementary figures and images for: Crystal structure of cyclo­hexyl­ammonium thio­cyanate
Source: Acta Crystallogr E Crystallogr Commun. 2015 Jan 1;71(Pt 1):o62–3. doi: 10.1107/S2056989014027297 (PMC4331923; doi:10.1107/S2056989014027297)

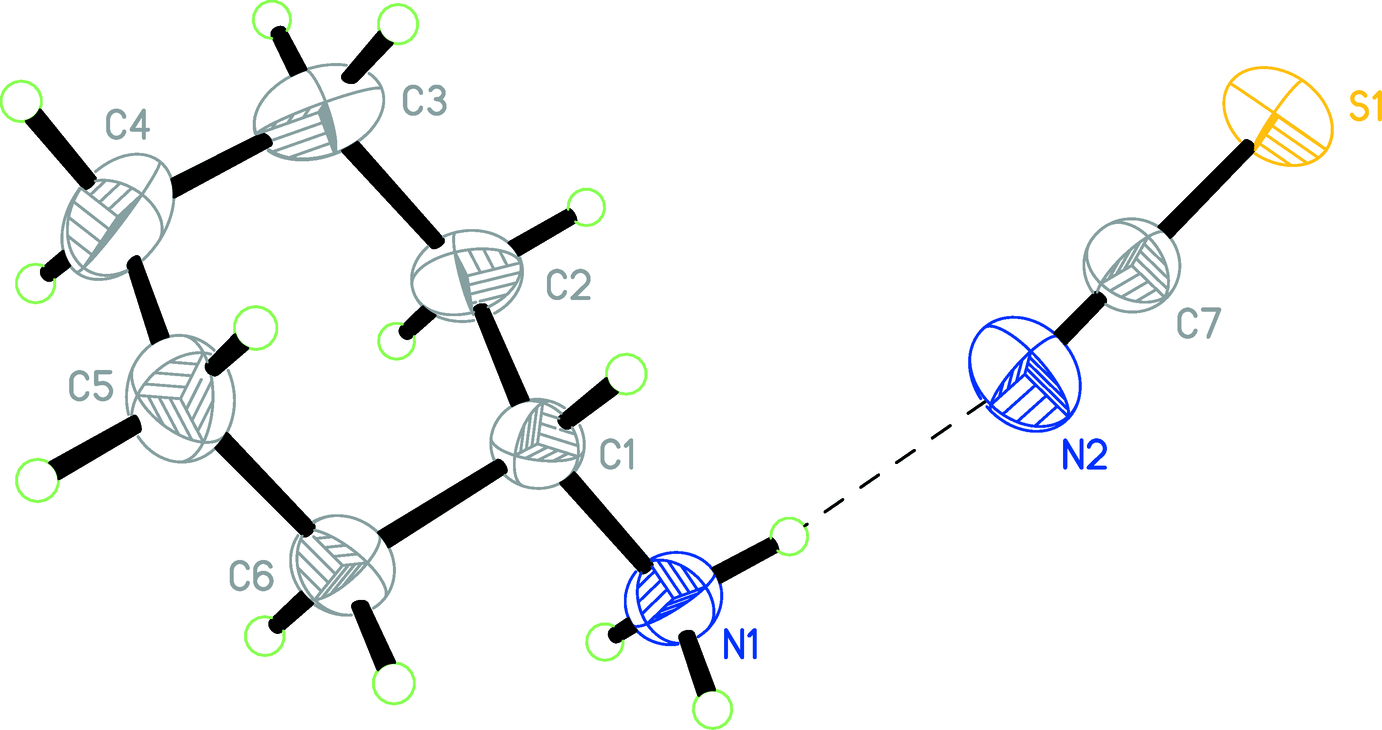

Supplement: Supplementary file 4 [file e-71-00o62-fig1.tif]

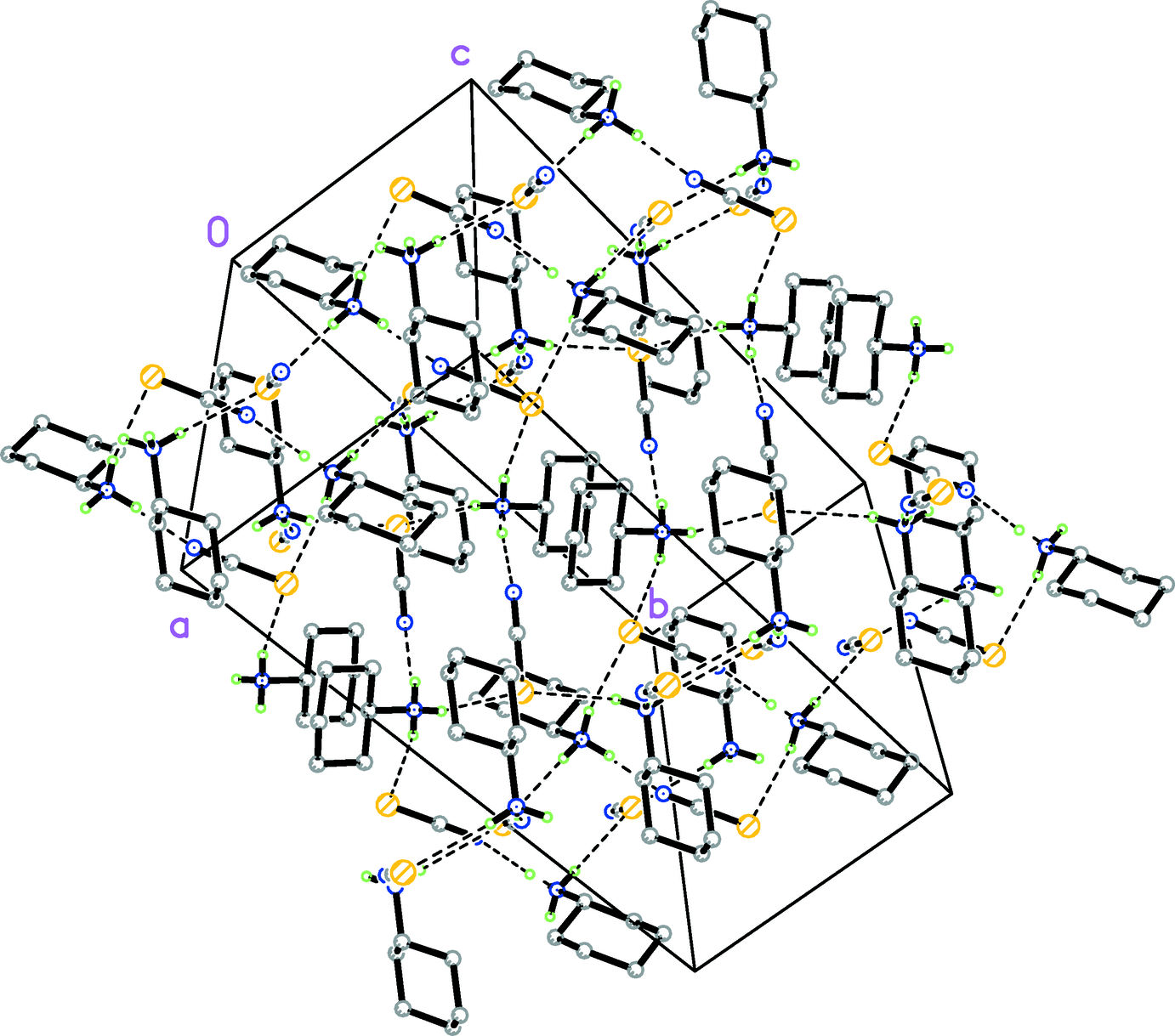

Supplement: Supplementary file 5 [file e-71-00o62-fig2.tif]
